# Poly[[aqua­(μ_2_-4,4′-bipyridine-κ^2^ *N*:*N*′)[μ_3_-3-bromo-2-(carboxyl­atometh­yl)­benzoato-κ^3^ *O* ^1^:*O* ^1′^:*O* ^2^]cadmium] monohydrate]

**DOI:** 10.1107/S1600536812031297

**Published:** 2012-07-14

**Authors:** Yangmei Liu, Kai Cao, Fenglin Wang

**Affiliations:** a National Food Packaging Products Quality Supervision and Inspection Center, Jiangsu Provincial Supervising and Testing Research Institute for Products Quality, Nanjing 210007, Jiangsu, People’s Republic of China; b School of Chemistry and Chemical Engineering, Nanjing University, Nanjing 210093, Jiangsu, People’s Republic of China

## Abstract

In the title compound, {[Cd(C_9_H_5_BrO_4_)(C_10_H_8_N_2_)(H_2_O)]·H_2_O}_*n*_, the Cd^II^ atom has a distorted octa­hedral coordination geometry. Two N atoms from two 4,4′-bipyridine (bipy) ligands occupy the axial positions, while the equatorial positions are furnished by three carboxyl­ate O atoms from three 3-bromo-2-(carboxyl­atometh­yl)benzoate (bcb) ligands and one O atom from a water mol­ecule. The bipy and bcb ligands link the Cd^II^ atoms into a three-dimensional network. O—H⋯O hydrogen bonds and π–π inter­actions between the pyridine and benzene rings [centroid–centroid distance = 3.736 (4) Å] are present in the crystal.

## Related literature

For related structures, see: Liu *et al.* (2010 ▶).
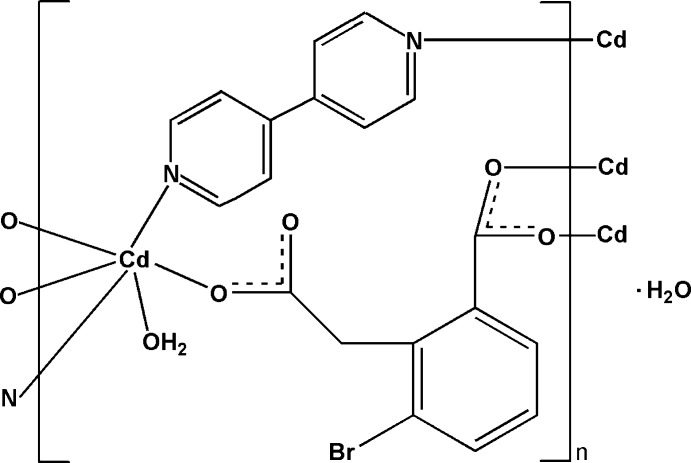


## Experimental

### 

#### Crystal data

[Cd(C_9_H_5_BrO_4_)(C_10_H_8_N_2_)(H_2_O)]·H_2_O
*M* *_r_* = 561.66
Monoclinic, 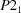

*a* = 9.3257 (19) Å
*b* = 9.1312 (18) Å
*c* = 11.652 (2) Å
β = 101.344 (4)°
*V* = 972.8 (3) Å^3^
*Z* = 2
Mo *K*α radiation
μ = 3.22 mm^−1^
*T* = 291 K
0.29 × 0.26 × 0.23 mm

#### Data collection

Bruker APEXII CCD diffractometer
Absorption correction: multi-scan (*SADABS*; Bruker, 2001 ▶) *T*_min_ = 0.466, *T*_max_ = 0.538
5248 measured reflections
3122 independent reflections
2807 reflections with *I* > 2σ(*I*)
*R*_int_ = 0.033

#### Refinement

*R*[*F* ^2^ > 2σ(*F* ^2^)] = 0.049
*wR*(*F* ^2^) = 0.118
*S* = 1.05
3122 reflections
251 parameters
1 restraint
H-atom parameters constrained
Δρ_max_ = 0.49 e Å^−3^
Δρ_min_ = −0.93 e Å^−3^
Absolute structure: Flack (1983 ▶), 1096 Friedel pairs
Flack parameter: 0.07 (2)

### 

Data collection: *APEX2* (Bruker, 2007 ▶); cell refinement: *SAINT* (Bruker, 2007 ▶); data reduction: *SAINT*; program(s) used to solve structure: *SHELXTL* (Sheldrick, 2008 ▶); program(s) used to refine structure: *SHELXTL*; molecular graphics: *XP* in *SHELXTL* and *DIAMOND* (Brandenburg, 1999 ▶); software used to prepare material for publication: *SHELXTL*.

## Supplementary Material

Crystal structure: contains datablock(s) I, global. DOI: 10.1107/S1600536812031297/hy2559sup1.cif

Structure factors: contains datablock(s) I. DOI: 10.1107/S1600536812031297/hy2559Isup2.hkl

Additional supplementary materials:  crystallographic information; 3D view; checkCIF report

## Figures and Tables

**Table 1:** Hydrogen-bond geometry (Å, °)

*D*—H⋯*A*	*D*—H	H⋯*A*	*D*⋯*A*	*D*—H⋯*A*
O5—H5*X*⋯O3^i^	0.85	2.47	2.963 (8)	118
O5—H5*Y*⋯O2^i^	0.85	2.22	2.731 (9)	119
O6—H6*X*⋯O1	0.85	2.51	3.201 (9)	139
O6—H6*Y*⋯O2	0.85	2.14	2.643 (9)	117

Symmetry code: (i) 
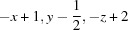
.

**Fig. 1.**
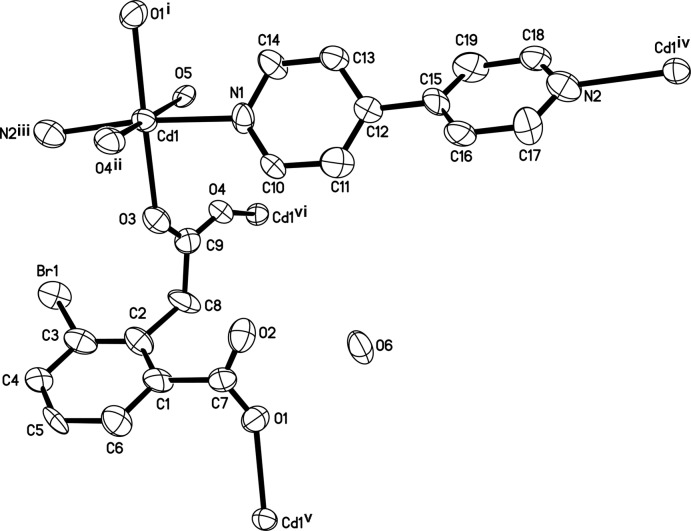
The aymmetric unit of the title compound, with 50% probability displacement ellipsoids. [Symmetry codes: (i) -1+x, y, z; (ii) 1-x, 1/2+y, 2-z; (iii) x, y, 1+z; (iv) x, y, -1+z; (v) 1+x, y, z; (vi) 1-x, -1/2+y, 2-z.]

**Fig. 2.**
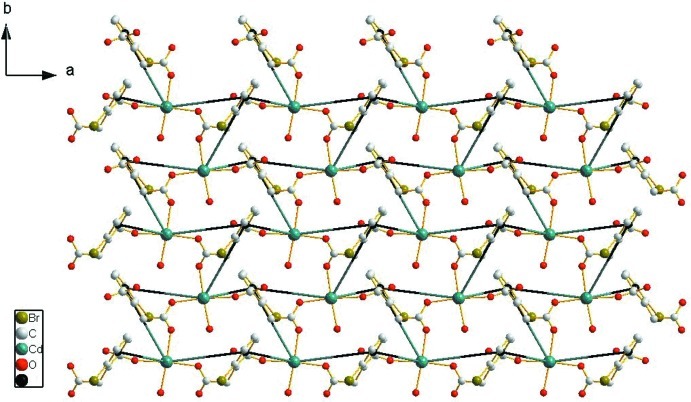
The two-dimensional polymeric layer in the ab plane, which can be simplified to a 63 network. Black balls denote the centroids of the benzene rings.

**Fig. 3.**
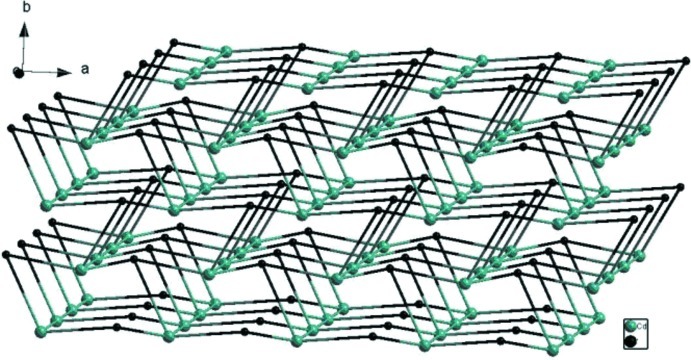
Schematic view of the three-dimensional network of the title compound. Black balls denote the centroids of the benzene rings.

## supplementary crystallographic information

### Comment

In the title complex (Fig. 1), the carboxylate group containing O1 and O2 is monocoordinated and the other carboxylate group containing O3 and O4 is bidentate and bridging. Therefore, the 3-bromo-2-(carboxymethyl)benzoate (bcb) ligand coordinates with three Cd atoms. The Cd atom is coordinated by three bcb ligands, forming a two-dimensional polymeric layer parallel to (1 1 0). Since each Cd atom is coordinated by three bcb ligands, from a topology viewpoint the Cd atom can be considered as a 3-connecting node and the center of benzene ring of the bcb ligand also acts as a 3-connecting node. In this way, the polymeric layer can be simplified to a 6^3^ network (Fig. 2). The 4,4'-bipyridine ligands act as bridges to connect the neighboring Cd atoms which come from different layers, forming a three-dimensional rigid porous network. This three-dimensional topology can be defined with Schläfli symbol (6^3^)(6^9^.8) (Fig. 3).

### Experimental

A mixture of 3-bromo-2-(carboxymethyl)benzoic acid (0.1 mmol, 25.9 mg), 4,4'-bipyridine (0.1 mmol, 19.5 mg), Cd(NO_3_)_2_.4H_2_O (0.1 mmol, 30.9 mg) and 8 ml water was sealed in a 23 ml Teflon-lined autoclave. The pH value of the mixture was adjusted to 7.0 with NaOH solution. The autoclave was kept at 393 K for 3 days. After the mixture was slowly cooled to room temperature, colorless crystals of the title compound were obtained.

### Refinement

H atoms bonded to C atoms were positioned geometrically and refined as riding atoms, with C—H = 0.93 (aromatic) and 0.97 (CH_2_) Å and with *U*_iso_(H) = 1.2*U*_eq_(C). Water H atoms were located in a difference Fourier map and refined as riding, with O—H = 0.85 Å and with *U*_iso_(H) = 1.2(1.5 for O5)*U*_eq_(O).

### Crystal data

**Table:**

[Cd(C_9_H_5_BrO_4_)(C_10_H_8_N_2_)(H_2_O)]·H_2_O	*F*(000) = 552
*M**_r_* = 561.66	*D*_x_ = 1.918 Mg m^−^^3^
Monoclinic, *P*2_1_	Mo *K*α radiation, λ = 0.71073 Å
Hall symbol: P 2yb	Cell parameters from 1319 reflections
*a* = 9.3257 (19) Å	θ = 2.6–21.3°
*b* = 9.1312 (18) Å	µ = 3.22 mm^−^^1^
*c* = 11.652 (2) Å	*T* = 291 K
β = 101.344 (4)°	Block, colorless
*V* = 972.8 (3) Å^3^	0.29 × 0.26 × 0.23 mm
*Z* = 2	

### Data collection

**Table:**

Bruker APEXII CCD diffractometer	3122 independent reflections
Radiation source: sealed tube	2807 reflections with *I* > 2σ(*I*)
Graphite monochromator	*R*_int_ = 0.033
φ and ω scans	θ_max_ = 26.0°, θ_min_ = 1.8°
Absorption correction: multi-scan (*SADABS*; Bruker, 2001)	*h* = −11→8
*T*_min_ = 0.466, *T*_max_ = 0.538	*k* = −11→10
5248 measured reflections	*l* = −14→13

### Refinement

**Table:**

Refinement on *F*^2^	Secondary atom site location: difference Fourier map
Least-squares matrix: full	Hydrogen site location: inferred from neighbouring sites
*R*[*F*^2^ > 2σ(*F*^2^)] = 0.049	H-atom parameters constrained
*wR*(*F*^2^) = 0.118	*w* = 1/[σ^2^(*F*_o_^2^) + (0.07*P*)^2^ + 1.99*P*] where *P* = (*F*_o_^2^ + 2*F*_c_^2^)/3
*S* = 1.05	(Δ/σ)_max_ < 0.001
3122 reflections	Δρ_max_ = 0.49 e Å^−^^3^
251 parameters	Δρ_min_ = −0.93 e Å^−^^3^
1 restraint	Absolute structure: Flack (1983), 1096 Friedel pairs
Primary atom site location: structure-invariant direct methods	Flack parameter: 0.07 (2)

### Special details

**Table:**

Geometry. All e.s.d.'s (except the e.s.d. in the dihedral angle between two l.s. planes) are estimated using the full covariance matrix. The cell e.s.d.'s are taken into account individually in the estimation of e.s.d.'s in distances, angles and torsion angles; correlations between e.s.d.'s in cell parameters are only used when they are defined by crystal symmetry. An approximate (isotropic) treatment of cell e.s.d.'s is used for estimating e.s.d.'s involving l.s. planes.
Refinement. Refinement of *F*^2^ against ALL reflections. The weighted *R*-factor *wR* and goodness of fit *S* are based on *F*^2^, conventional *R*-factors *R* are based on *F*, with *F* set to zero for negative *F*^2^. The threshold expression of *F*^2^ > σ(*F*^2^) is used only for calculating *R*-factors(gt) *etc*. and is not relevant to the choice of reflections for refinement. *R*-factors based on *F*^2^ are statistically about twice as large as those based on *F*, and *R*- factors based on ALL data will be even larger.

### Fractional atomic coordinates and isotropic or equivalent isotropic displacement parameters (Å^2^)

**Table:**

	*x*	*y*	*z*	*U*_iso_*/*U*_eq_	
Br1	0.78647 (11)	0.15671 (12)	1.36696 (9)	0.0477 (3)	
C1	0.9844 (6)	0.3672 (6)	1.1234 (3)	0.038 (2)	
C2	0.9014 (6)	0.2688 (5)	1.1738 (4)	0.040 (2)	
C3	0.8909 (6)	0.2844 (6)	1.2905 (4)	0.043 (2)	
C4	0.9634 (6)	0.3984 (7)	1.3568 (3)	0.0325 (18)	
H4	0.9595	0.4095	1.4322	0.039*	
C5	1.0464 (6)	0.4968 (6)	1.3064 (4)	0.037 (2)	
H5	1.0956	0.5725	1.3513	0.045*	
C6	1.0569 (6)	0.4812 (6)	1.1896 (4)	0.044 (2)	
H6	1.1103	0.5453	1.1582	0.053*	
C7	0.9925 (10)	0.3554 (11)	0.9975 (8)	0.043 (2)	
C8	0.8249 (10)	0.1429 (14)	1.0972 (8)	0.048 (2)	
H8A	0.8339	0.0584	1.1486	0.058*	
H8B	0.8795	0.1215	1.0365	0.058*	
C9	0.6697 (10)	0.1577 (12)	1.0473 (8)	0.039 (2)	
C10	0.4900 (9)	0.3415 (11)	0.7781 (7)	0.042 (3)	
H10	0.5714	0.3723	0.8322	0.050*	
C11	0.4933 (11)	0.3408 (11)	0.6554 (8)	0.047 (3)	
H11	0.5784	0.3712	0.6321	0.056*	
C12	0.3785 (8)	0.2859 (10)	0.5800 (7)	0.0316 (17)	
C13	0.2515 (10)	0.2495 (11)	0.6235 (8)	0.040 (2)	
H13	0.1689	0.2156	0.5723	0.048*	
C14	0.2516 (11)	0.2538 (12)	0.7434 (7)	0.043 (2)	
H14	0.1700	0.2206	0.7705	0.051*	
C15	0.3746 (8)	0.2923 (14)	0.4498 (6)	0.039 (2)	
C16	0.4478 (10)	0.4038 (11)	0.4039 (8)	0.040 (2)	
H16	0.5014	0.4746	0.4514	0.048*	
C17	0.4438 (11)	0.4024 (11)	0.2825 (8)	0.043 (2)	
H17	0.4953	0.4739	0.2506	0.051*	
C18	0.2976 (10)	0.2033 (13)	0.2613 (9)	0.048 (3)	
H18	0.2452	0.1331	0.2123	0.057*	
C19	0.3039 (11)	0.1901 (11)	0.3853 (9)	0.046 (2)	
H19	0.2529	0.1157	0.4142	0.055*	
Cd1	0.35516 (6)	0.30525 (7)	1.01349 (5)	0.03236 (17)	
N1	0.3714 (7)	0.3032 (12)	0.8186 (5)	0.0387 (15)	
N2	0.3662 (8)	0.3106 (13)	0.2123 (6)	0.0440 (16)	
O1	1.1007 (6)	0.3167 (10)	0.9680 (5)	0.0450 (15)	
O2	0.8816 (8)	0.3873 (8)	0.9228 (6)	0.0454 (16)	
O3	0.6015 (7)	0.2669 (7)	1.0621 (5)	0.0374 (15)	
O4	0.6104 (7)	0.0555 (8)	0.9904 (5)	0.0385 (15)	
O5	0.3078 (7)	0.0660 (7)	0.9920 (6)	0.0367 (15)	
H5Y	0.2159	0.0524	0.9757	0.055*	
H5X	0.3462	0.0320	0.9372	0.055*	
O6	0.9272 (8)	0.3735 (8)	0.7062 (5)	0.0464 (17)	
H6X	0.9963	0.3969	0.7624	0.056*	
H6Y	0.8510	0.3716	0.7360	0.056*	

### Atomic displacement parameters (Å^2^)

**Table:**

	*U*^11^	*U*^22^	*U*^33^	*U*^12^	*U*^13^	*U*^23^
Br1	0.0485 (6)	0.0441 (6)	0.0474 (5)	−0.0073 (4)	0.0018 (4)	0.0159 (5)
C1	0.029 (4)	0.049 (5)	0.030 (4)	0.003 (3)	−0.008 (3)	0.013 (4)
C2	0.048 (5)	0.026 (5)	0.042 (4)	−0.005 (3)	−0.003 (4)	−0.002 (4)
C3	0.037 (4)	0.037 (6)	0.047 (4)	0.002 (4)	−0.010 (3)	0.010 (4)
C4	0.030 (4)	0.037 (5)	0.026 (4)	0.006 (3)	−0.004 (3)	0.012 (4)
C5	0.042 (4)	0.031 (5)	0.032 (4)	−0.008 (4)	−0.010 (4)	−0.014 (4)
C6	0.049 (5)	0.048 (6)	0.033 (4)	−0.002 (4)	−0.001 (4)	0.008 (4)
C7	0.037 (5)	0.052 (6)	0.042 (5)	−0.022 (4)	0.014 (4)	−0.004 (4)
C8	0.036 (5)	0.064 (7)	0.037 (5)	−0.013 (5)	−0.014 (4)	0.009 (5)
C9	0.037 (4)	0.043 (5)	0.040 (5)	−0.006 (4)	0.011 (4)	−0.004 (5)
C10	0.032 (4)	0.059 (8)	0.032 (4)	−0.010 (4)	−0.001 (3)	−0.016 (4)
C11	0.045 (5)	0.049 (7)	0.045 (5)	−0.005 (4)	0.007 (4)	0.018 (5)
C12	0.034 (4)	0.023 (5)	0.036 (4)	−0.002 (3)	0.002 (3)	0.003 (4)
C13	0.031 (4)	0.044 (5)	0.042 (5)	−0.004 (4)	0.002 (4)	−0.006 (4)
C14	0.046 (5)	0.055 (6)	0.025 (4)	−0.022 (4)	0.001 (4)	0.014 (4)
C15	0.035 (4)	0.051 (6)	0.026 (3)	0.021 (5)	−0.007 (3)	0.006 (5)
C16	0.030 (4)	0.043 (6)	0.046 (5)	−0.013 (4)	0.001 (4)	−0.012 (4)
C17	0.064 (6)	0.032 (5)	0.035 (4)	−0.025 (4)	0.014 (4)	0.004 (4)
C18	0.029 (4)	0.063 (7)	0.050 (6)	−0.011 (4)	0.004 (4)	−0.019 (5)
C19	0.044 (5)	0.037 (5)	0.057 (6)	−0.018 (4)	0.008 (4)	0.013 (5)
Cd1	0.0292 (3)	0.0353 (3)	0.0319 (3)	−0.0075 (3)	0.00435 (19)	−0.0014 (3)
N1	0.048 (4)	0.038 (4)	0.033 (3)	−0.019 (5)	0.016 (3)	−0.013 (5)
N2	0.043 (4)	0.038 (4)	0.046 (4)	0.002 (5)	−0.004 (3)	0.005 (5)
O1	0.038 (3)	0.049 (4)	0.052 (3)	−0.007 (4)	0.017 (3)	−0.013 (4)
O2	0.054 (4)	0.044 (4)	0.041 (3)	0.011 (3)	0.015 (3)	0.000 (3)
O3	0.041 (3)	0.035 (4)	0.034 (3)	−0.003 (3)	0.000 (2)	0.003 (3)
O4	0.032 (3)	0.048 (4)	0.033 (3)	−0.004 (3)	−0.001 (3)	−0.008 (3)
O5	0.026 (3)	0.037 (4)	0.048 (3)	0.014 (3)	0.007 (3)	−0.019 (3)
O6	0.060 (4)	0.048 (4)	0.029 (3)	−0.015 (3)	0.004 (3)	−0.008 (3)

### Geometric parameters (Å, º)

**Table:**

Br1—C3	1.855 (4)	C13—H13	0.9299
C1—C2	1.3900	C14—N1	1.355 (11)
C1—C6	1.3900	C14—H14	0.9299
C1—C7	1.487 (10)	C15—C19	1.294 (15)
C2—C3	1.3900	C15—C16	1.390 (15)
C2—C8	1.541 (11)	C16—C17	1.408 (13)
C3—C4	1.3900	C16—H16	0.9300
C4—C5	1.3900	C17—N2	1.290 (13)
C4—H4	0.8922	C17—H17	0.9300
C5—C6	1.3900	C18—N2	1.356 (15)
C5—H5	0.9318	C18—C19	1.440 (15)
C6—H6	0.8918	C18—H18	0.9300
C7—O1	1.182 (11)	C19—H19	0.9300
C7—O2	1.248 (12)	Cd1—O5	2.233 (7)
C8—C9	1.455 (13)	Cd1—O3	2.282 (6)
C8—H8A	0.9700	Cd1—N2^i^	2.298 (7)
C8—H8B	0.9700	Cd1—N1	2.305 (6)
C9—O4	1.213 (12)	Cd1—O4^ii^	2.309 (8)
C9—O3	1.213 (12)	Cd1—O1^iii^	2.330 (6)
C10—N1	1.332 (11)	N2—Cd1^iv^	2.298 (7)
C10—C11	1.435 (13)	O1—Cd1^v^	2.330 (6)
C10—H10	0.9300	O4—Cd1^vi^	2.309 (8)
C11—C12	1.341 (13)	O5—H5Y	0.8500
C11—H11	0.9299	O5—H5X	0.8500
C12—C13	1.417 (12)	O6—H6X	0.8500
C12—C15	1.511 (11)	O6—H6Y	0.8500
C13—C14	1.396 (13)		
			
C2—C1—C6	120.0	C13—C14—H14	119.4
C2—C1—C7	120.7 (5)	C19—C15—C16	122.5 (8)
C6—C1—C7	119.2 (5)	C19—C15—C12	117.7 (9)
C1—C2—C3	120.0	C16—C15—C12	119.8 (9)
C1—C2—C8	118.1 (5)	C15—C16—C17	117.5 (8)
C3—C2—C8	121.9 (5)	C15—C16—H16	121.9
C4—C3—C2	120.0	C17—C16—H16	120.6
C4—C3—Br1	116.6 (3)	N2—C17—C16	123.0 (8)
C2—C3—Br1	123.4 (3)	N2—C17—H17	118.1
C3—C4—C5	120.0	C16—C17—H17	118.8
C3—C4—H4	121.5	N2—C18—C19	123.6 (9)
C5—C4—H4	118.5	N2—C18—H18	118.3
C6—C5—C4	120.0	C19—C18—H18	118.1
C6—C5—H5	120.3	C15—C19—C18	116.0 (9)
C4—C5—H5	119.7	C15—C19—H19	123.7
C5—C6—C1	120.0	C18—C19—H19	120.1
C5—C6—H6	119.1	O5—Cd1—O3	92.6 (2)
C1—C6—H6	120.9	O5—Cd1—N2^i^	95.9 (3)
O1—C7—O2	120.3 (9)	O3—Cd1—N2^i^	84.8 (2)
O1—C7—C1	121.3 (9)	O5—Cd1—N1	86.1 (3)
O2—C7—C1	118.4 (8)	O3—Cd1—N1	89.1 (2)
C9—C8—C2	118.2 (10)	N2^i^—Cd1—N1	173.8 (2)
C9—C8—H8A	106.2	O5—Cd1—O4^ii^	172.3 (2)
C2—C8—H8A	105.2	O3—Cd1—O4^ii^	91.1 (2)
C9—C8—H8B	110.5	N2^i^—Cd1—O4^ii^	91.2 (3)
C2—C8—H8B	108.9	N1—Cd1—O4^ii^	87.3 (3)
H8A—C8—H8B	107.2	O5—Cd1—O1^iii^	81.3 (3)
O4—C9—O3	120.9 (9)	O3—Cd1—O1^iii^	173.6 (3)
O4—C9—C8	117.2 (10)	N2^i^—Cd1—O1^iii^	94.0 (2)
O3—C9—C8	121.8 (10)	N1—Cd1—O1^iii^	92.1 (2)
N1—C10—C11	122.2 (8)	O4^ii^—Cd1—O1^iii^	95.2 (3)
N1—C10—H10	117.6	C10—N1—C14	119.6 (7)
C11—C10—H10	120.2	C10—N1—Cd1	124.5 (5)
C12—C11—C10	118.9 (8)	C14—N1—Cd1	115.8 (5)
C12—C11—H11	122.5	C17—N2—C18	117.0 (8)
C10—C11—H11	118.4	C17—N2—Cd1^iv^	124.2 (7)
C11—C12—C13	117.9 (8)	C18—N2—Cd1^iv^	118.5 (7)
C11—C12—C15	120.2 (8)	C7—O1—Cd1^v^	146.8 (6)
C13—C12—C15	120.7 (7)	C9—O3—Cd1	128.4 (6)
C14—C13—C12	121.0 (8)	C9—O4—Cd1^vi^	136.2 (7)
C14—C13—H13	119.2	Cd1—O5—H5Y	109.7
C12—C13—H13	119.6	Cd1—O5—H5X	109.7
N1—C14—C13	119.7 (8)	H5Y—O5—H5X	109.5
N1—C14—H14	120.9	H6X—O6—H6Y	105.0
			
C6—C1—C2—C3	0.0	C19—C15—C16—C17	−0.3 (15)
C7—C1—C2—C3	−177.8 (7)	C12—C15—C16—C17	178.4 (9)
C6—C1—C2—C8	−179.2 (7)	C15—C16—C17—N2	5.9 (16)
C7—C1—C2—C8	3.1 (8)	C16—C15—C19—C18	−3.8 (15)
C1—C2—C3—C4	0.0	C12—C15—C19—C18	177.5 (8)
C8—C2—C3—C4	179.1 (7)	N2—C18—C19—C15	3.3 (16)
C1—C2—C3—Br1	−178.6 (5)	C11—C10—N1—C14	3.9 (17)
C8—C2—C3—Br1	0.5 (7)	C11—C10—N1—Cd1	−178.8 (8)
C2—C3—C4—C5	0.0	C13—C14—N1—C10	−2.8 (17)
Br1—C3—C4—C5	178.7 (4)	C13—C14—N1—Cd1	179.7 (8)
C3—C4—C5—C6	0.0	O5—Cd1—N1—C10	−120.4 (10)
C4—C5—C6—C1	0.0	O3—Cd1—N1—C10	−27.7 (10)
C2—C1—C6—C5	0.0	O4^ii^—Cd1—N1—C10	63.4 (10)
C7—C1—C6—C5	177.8 (7)	O1^iii^—Cd1—N1—C10	158.5 (10)
C2—C1—C7—O1	−109.3 (9)	O5—Cd1—N1—C14	57.1 (9)
C6—C1—C7—O1	73.0 (11)	O3—Cd1—N1—C14	149.7 (9)
C2—C1—C7—O2	70.6 (10)	O4^ii^—Cd1—N1—C14	−119.1 (9)
C6—C1—C7—O2	−107.1 (8)	O1^iii^—Cd1—N1—C14	−24.0 (9)
C1—C2—C8—C9	−100.5 (9)	C16—C17—N2—C18	−6.4 (16)
C3—C2—C8—C9	80.4 (9)	C16—C17—N2—Cd1^iv^	179.6 (8)
C2—C8—C9—O4	−177.7 (8)	C19—C18—N2—C17	1.8 (16)
C2—C8—C9—O3	1.9 (14)	C19—C18—N2—Cd1^iv^	176.2 (8)
N1—C10—C11—C12	−6.9 (16)	O2—C7—O1—Cd1^v^	151.1 (10)
C10—C11—C12—C13	8.3 (14)	C1—C7—O1—Cd1^v^	−29 (2)
C10—C11—C12—C15	176.2 (9)	O4—C9—O3—Cd1	8.0 (13)
C11—C12—C13—C14	−7.6 (14)	C8—C9—O3—Cd1	−171.6 (6)
C15—C12—C13—C14	−175.3 (9)	O5—Cd1—O3—C9	16.6 (8)
C12—C13—C14—N1	4.6 (16)	N2^i^—Cd1—O3—C9	112.2 (8)
C11—C12—C15—C19	150.4 (10)	N1—Cd1—O3—C9	−69.5 (8)
C13—C12—C15—C19	−42.1 (13)	O4^ii^—Cd1—O3—C9	−156.7 (8)
C11—C12—C15—C16	−28.3 (13)	O3—C9—O4—Cd1^vi^	−152.0 (7)
C13—C12—C15—C16	139.1 (9)	C8—C9—O4—Cd1^vi^	27.7 (13)

Symmetry codes: (i) *x*, *y*, *z*+1; (ii) −*x*+1, *y*+1/2, −*z*+2; (iii) *x*−1, *y*, *z*; (iv) *x*, *y*, *z*−1; (v) *x*+1, *y*, *z*; (vi) −*x*+1, *y*−1/2, −*z*+2.

### Hydrogen-bond geometry (Å, º)

**Table:**

*D*—H···*A*	*D*—H	H···*A*	*D*···*A*	*D*—H···*A*
O5—H5*X*···O3^vi^	0.85	2.47	2.963 (8)	118
O5—H5*Y*···O2^vi^	0.85	2.22	2.731 (9)	119
O6—H6*X*···O1	0.85	2.51	3.201 (9)	139
O6—H6*Y*···O2	0.85	2.14	2.643 (9)	117

Symmetry code: (vi) −*x*+1, *y*−1/2, −*z*+2.
